# Bariatric Surgery as an Adjunctive Treatment for Failed Back Surgery Syndrome: A Case Report Highlighting a Multidisciplinary Approach

**DOI:** 10.7759/cureus.79331

**Published:** 2025-02-19

**Authors:** Richelle Huey Bing Chua, Guo Hou Loo, Nani HL, Guhan Muthkumaran, Nik Ritza Kosai

**Affiliations:** 1 Upper GI and Metabolic Surgery Unit, Department of Surgery, Faculty of Medicine, The National University of Malaysia, Kuala Lumpur, MYS; 2 Upper GI and Metabolic Surgery Unit, Department of Surgery, Universiti Kebangsaan Malaysia Medical Centre, Kuala Lumpur, MYS; 3 Breast and Endocrine Surgery, School of Medical and Life Sciences, Sunway University, Kuala Lumpur, MYS

**Keywords:** asian population, chronic back pain, metabolic surgery, obesity, stimulator pulse generator

## Abstract

Failed back surgery syndrome (FBSS) poses a significant challenge in chronic pain management, particularly in patients with obesity, where excess weight exacerbates spinal strain and pain. Bariatric surgery has been explored as a potential adjunctive treatment for pain relief by reducing mechanical stress and systemic inflammation. We report the case of a 42-year-old woman with a history of FBSS, chronic back pain, narcolepsy, rheumatoid arthritis, and obesity, who underwent laparoscopic sleeve gastrectomy as part of a multidisciplinary pain management strategy. Despite having a spinal cord stimulator for pain control, she remained dependent on opioids, gabapentin, and amitriptyline. Following an uneventful surgery, postoperative pain was effectively managed with a ketamine infusion, leading to significant pain score reduction. The patient was discharged with an optimized pain regimen and demonstrated early improvements in mobility and overall well-being. This report highlights the potential role of bariatric surgery in managing chronic pain in patients with FBSS and obesity. It underscores the importance of a multidisciplinary approach, preoperative planning, and tailored postoperative pain management in optimizing outcomes. Further research is warranted to evaluate the long-term impact of bariatric surgery on chronic pain management and functional recovery in this patient population.

## Introduction

Chronic low back pain (LBP) is a prevalent condition worldwide, affecting approximately 10% of the global population. It has significant adverse effects on physical functioning, limiting both the extent and complexity of physical activity [1-3]. Obesity is a major contributing factor, with one-third of the world population classified as overweight and 20% considered obese [4]. Currently, 500 million adults worldwide are affected by obesity, which is closely linked to musculoskeletal pain and physical dysfunction. The severity and incidence of chronic pain, including LBP, increase with higher BMI [4]. Obesity-related chronic pain encompasses a range of conditions, including joint and musculoskeletal pain, headaches, abdominal pain, pelvic pain, and neuropathic pain [4,5].

Studies indicate that up to 84% of individuals will experience at least one episode of LBP in their lifetime [6]. Management strategies for chronic LBP include pharmacological treatments such as oral non-steroidal anti-inflammatory drugs (NSAIDs) and topical lidocaine, spinal steroid injections, physiotherapy, psychological interventions, and multidisciplinary rehabilitation [7]. However, some patients experience persistent pain despite surgical intervention, a condition known as failed back surgery syndrome (FBSS). Bariatric surgery has been shown to provide significant weight loss, which may alleviate pain by reducing mechanical strain on the spine [8]. We present a case of a patient with chronic back pain, FBSS, and multiple comorbidities who underwent bariatric surgery to manage her weight and alleviate pain. This case has been reported according to the Surgical CAse REport (SCARE) guidelines [9].

## Case presentation

A 42-year-old female of Asian descent, weighing 85 kg with a BMI of 36 kg/m², presented with a 20-year history of chronic LBP secondary to L5/S1 spondylolisthesis. She had undergone spinal fusion surgery 15 years earlier but continued to experience persistent pain and functional impairment, leading to a diagnosis of FBSS. Her medical history was significant for narcolepsy, rheumatoid arthritis, hypertension, and gastroesophageal reflux disease (GERD). She also had a history of depression and anxiety, further complicating her chronic pain management. Over the years, she had developed opioid dependence due to escalating pain requiring long-term analgesic therapy.

Despite undergoing regular epidural and sacroiliac joint blocks for over a decade, her pain had remained refractory to conservative management. In an effort to improve her quality of life, a spinal cord stimulator (RestoreSensor™ SureScan™, Medtronic, Minneapolis, MN) was implanted. However, neuromodulation therapy had only provided partial pain relief, and she had continued to rely on multiple analgesics, including opioids (tramadol), gabapentin, and amitriptyline.

She reported significant limitations in daily activities, with difficulty in prolonged standing, walking, and climbing stairs. Her pain was exacerbated by physical exertion and only minimally relieved by medications. Given her progressive weight gain and increasing BMI, the mechanical burden on her spine was hypothesized to be a key factor exacerbating her symptoms. After multidisciplinary team discussions including input from spine surgeons, pain specialists, and bariatric surgeons, a decision was made to proceed with laparoscopic sleeve gastrectomy (LSG) to facilitate weight loss and potentially improve her pain and mobility.

**Figure 1:**
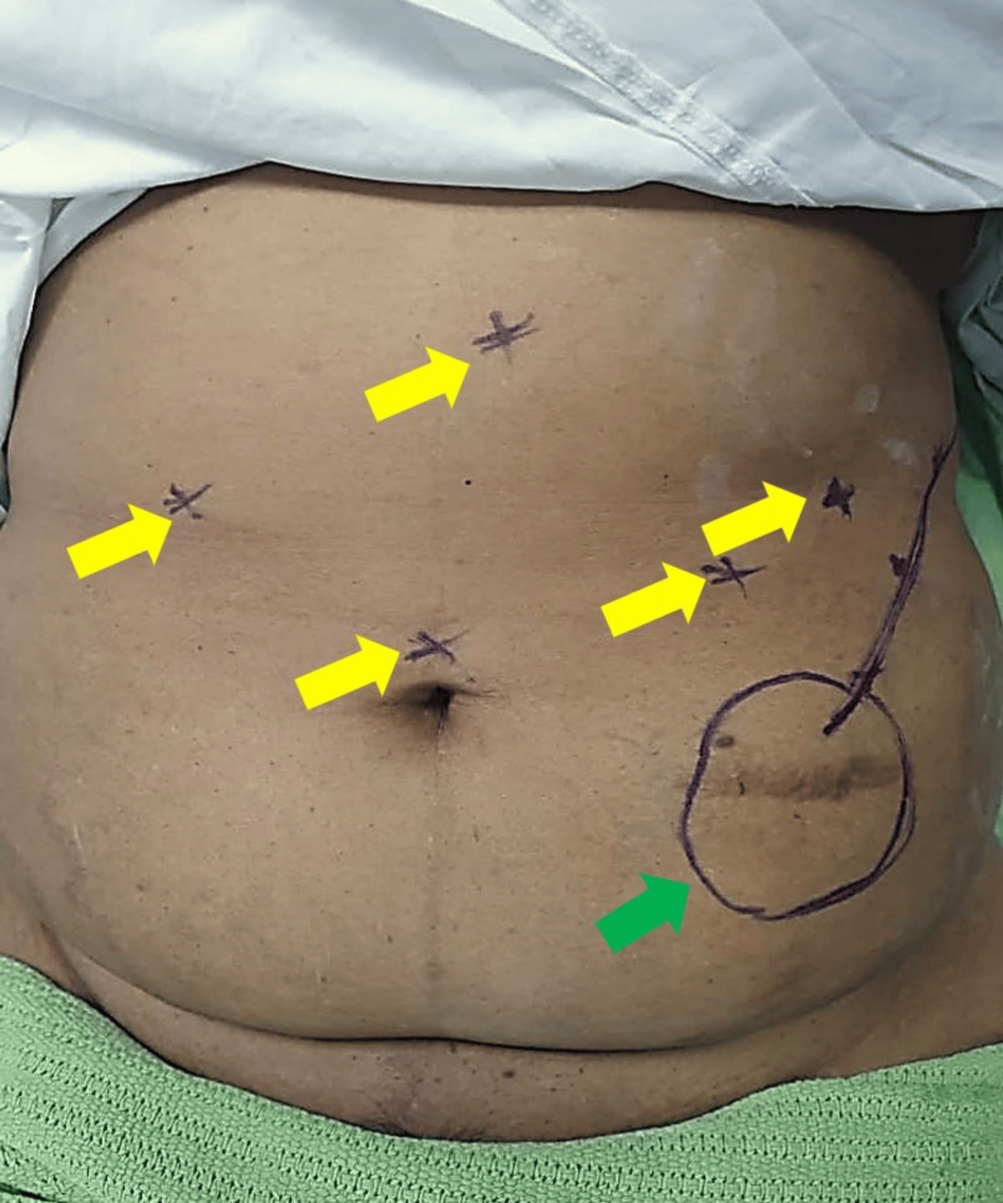
Intraoperative image of patient's abdomen - 1 The yellow arrows indicate proposed port placement sites and the green arrow depicts the location of the stimulator pulse generator

**Figure 2:**
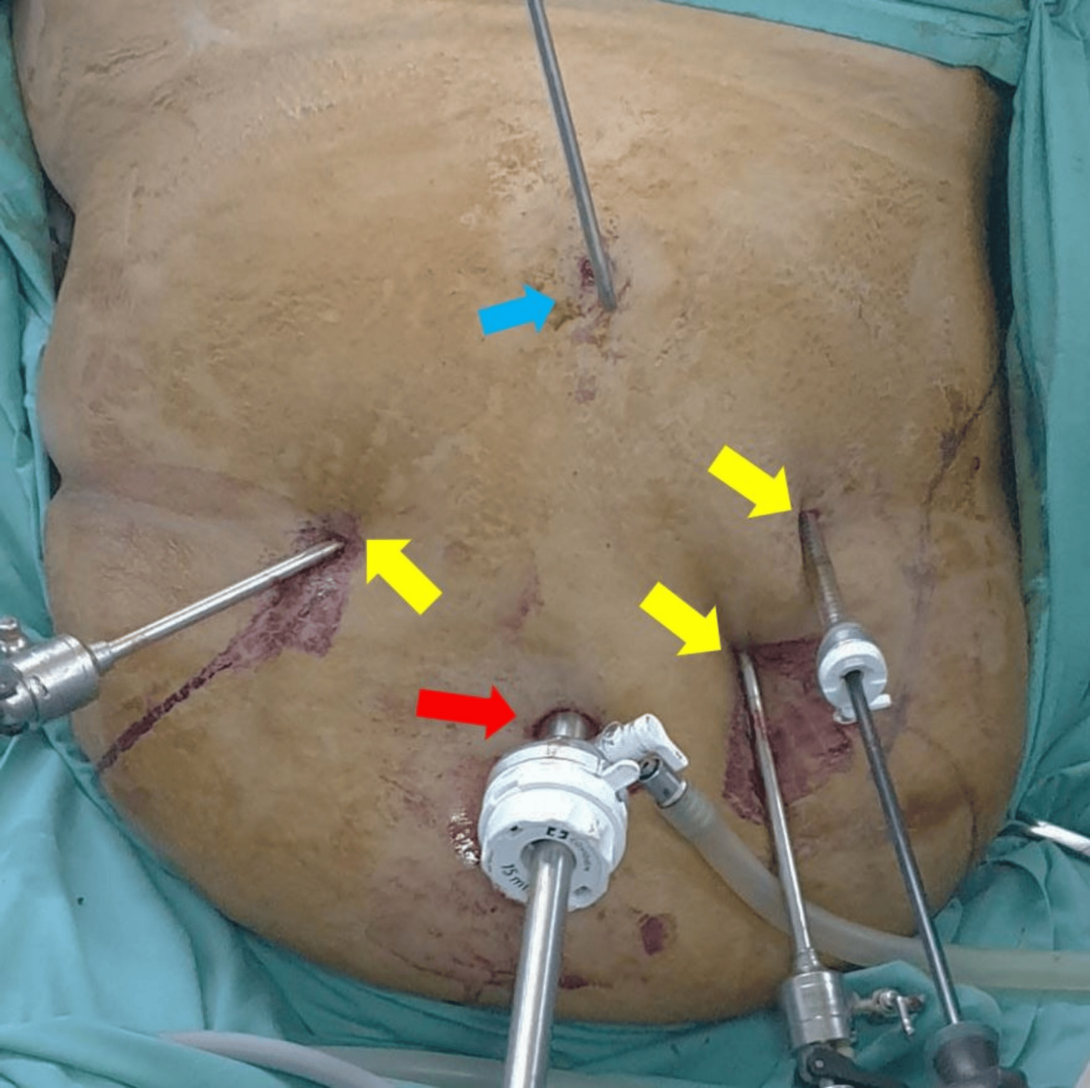
Intraoperative image of patient's abdomen - 2 The blue arrow indicates the position of the Nathanson liver retractor; the yellow arrows indicate the position of 5mm working ports; and the red arrow indicates the position of the 12mm camera port

A comprehensive preoperative evaluation was performed, including an MRI of the lumbosacral spine, confirming stable post-fusion changes with residual foraminal stenosis, venous Doppler ultrasound, ruling out deep vein thrombosis (DVT), and chronic venous insufficiency as well as cardiopulmonary assessment, ensuring fitness for laparoscopic surgery. Given her implanted spinal cord stimulator, special precautions were taken, including ultrasound-guided localization of the stimulator pulse generator to ensure safe port placement, and modification of trocar positioning to avoid interference with the device (Figures 1, 2).

**Figure 3:**
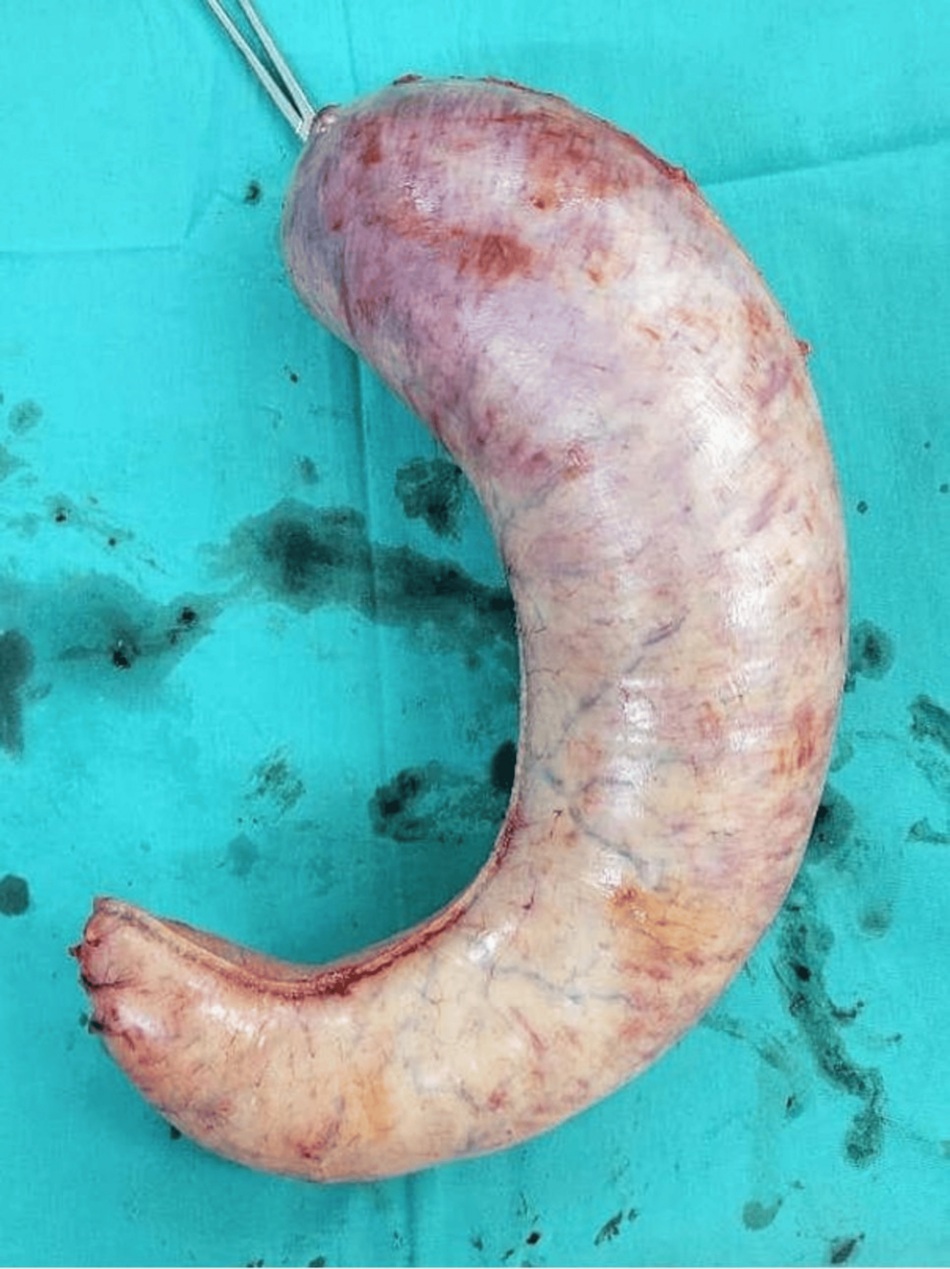
Specimen of resected portion of stomach (approximately 70% of the stomach)

LSG was performed successfully, with no intraoperative complications. The intraoperative assessment confirmed complete gastric resection with no staple line leakage (Figure 3).

In the immediate postoperative period, the patient was closely monitored in the post-anesthesia care unit (PACU). A ketamine infusion was initiated as part of a multimodal pain management strategy. She remained hemodynamically stable and was later transferred to the regular ward, where her pain was effectively controlled with a regimen of regular paracetamol, gabapentin, celecoxib, and etoricoxib. Granisetron was given as needed for the control of nausea.

By the time of discharge, the patient reported a pain score of 2/10 and was sent home with a structured pain management plan, including paracetamol, gabapentin, and celecoxib, along with scheduled follow-up appointments. At her three-year follow-up, she had achieved significant weight loss, with her BMI constant at 22 kg/m². She reported marked improvement in her chronic back pain, with increased mobility and reduced reliance on analgesics, demonstrating the potential role of bariatric surgery as an adjunct in managing FBSS.

## Discussion

Chronic back pain is a complex and multifactorial condition, particularly challenging in patients with obesity and multiple comorbidities. While conventional treatment options include pharmacologic therapy, physiotherapy, and neuromodulation, bariatric surgery has emerged as a potential adjunct therapy for obese patients with refractory pain [4]. We described the case of a 42-year-old woman with FBSS who underwent LSG as part of a multidisciplinary pain management strategy. FBSS refers to persistent or recurrent pain following spinal surgery, often necessitating complex pain management strategies. Following L5/S1 spinal fusion, our patient continued to experience severe pain [preoperative score: 8/10 on visual analog scale (VAS)], despite long-term opioid therapy and neuromodulation. Obesity is a known risk factor for increased postoperative complications in spinal surgery, including wound infections, hardware failure, and poor fusion rates [10]. Studies report a 16% complication rate in obese patients undergoing revision lumbar fusion and a 13.4% rate in primary lumbar fusion cases [11]. 

The incidence of FBSS is estimated at 80,000 new cases annually [12], and the success rate of repeated lumbar spine surgeries declines significantly with each subsequent operation [13]. Despite advances in surgical techniques, 40% of patients continue to experience chronic pain postoperatively [13]. Given these statistics, a multidimensional approach incorporating medical therapy, neuromodulation, and weight optimization is essential. Neuromodulatory interventions, such as spinal cord stimulation (SCS), have demonstrated efficacy in FBSS pain relief [11]. However, pain relief is often partial, as observed in our patient, who continued to require opioids, gabapentin, and amitriptyline despite the stimulator implantation. This highlights the need for complementary interventions, including weight management, to optimize outcomes.

While bariatric surgery does not provide immediate pain relief, it offers long-term benefits by reducing mechanical strain on the spine and improving overall metabolic health [4]. Multiple studies have demonstrated that post-bariatric weight loss correlates with reduced musculoskeletal pain, improved physical function, and better quality of life [14]. Our patient’s BMI decreased from 36 kg/m² to 22 kg/m² over three years. Concurrently, she reported improved pain control, with VAS scores decreasing from 8/10 (preoperative) to 2/10 (immediate postoperative, due to ketamine use), and stabilizing at 3/10 at the three-year follow-up. A reduction in analgesic requirements, where the patient currently takes gabapentin 900 mg once daily and tramadol 50 mg twice daily, compared to her preoperative regimen of high-dose opioids, gabapentin, and amitriptyline was also evident. She has also improved mobility, with increased tolerance for daily activities and exercise.

Pain control in the immediate postoperative period must be distinguished from long-term pain relief due to weight loss. Our patient reported a VAS score of 2/10 at discharge; however, this cannot be attributed to weight reduction, as significant weight loss occurs gradually over months. Instead, the use of multimodal analgesia including ketamine infusion, played a key role in her early pain control. According to the Canadian Consensus Statement on Pain Management after Bariatric Surgery, opioid-sparing strategies, including NMDA receptor antagonists like ketamine, are strongly recommended [13]. Ketamine not only provides effective acute pain relief but also prevents central sensitization, which is crucial for patients with chronic neuropathic pain and opioid dependence [8].

While the patient’s pain improved significantly, she still requires daily gabapentin and intermittent tramadol, emphasizing that bariatric surgery alone is not a definitive cure for FBSS. Rather, it serves as an adjunct to a broader pain management strategy. Future randomized controlled trials are warranted to evaluate the long-term efficacy of bariatric surgery in patients with FBSS. Standardized quantitative assessments, such as the Oswestry Disability Index (ODI) and SF-36 pain scores, could provide more objective measures of postoperative improvement.

## Conclusions

This report highlights the multifaceted nature of FBSS management, demonstrating that bariatric surgery, when integrated into a multimodal approach, can contribute to sustained pain reduction and improved functionality. Further research is needed to define the optimal patient selection criteria and the long-term benefits of metabolic surgery in chronic pain management.
